# Aging is a weak but relentless determinant of dementia severity

**DOI:** 10.18632/oncotarget.7759

**Published:** 2016-02-26

**Authors:** Donald R. Royall, Raymond F. Palmer

**Affiliations:** ^1^ Department of Psychiatry, The University of Texas Health Science Center, San Antonio, TX, USA; ^2^ Department of Medicine, The University of Texas Health Science Center, San Antonio, TX, USA; ^3^ Department of Family and Community Medicine, The University of Texas Health Science Center, San Antonio, TX, USA; ^4^ The South Texas Veterans' Health System, Audie L. Murphy Division GRECC, San Antonio, TX, USA

**Keywords:** aging, intelligence, dementia, senility, Gerotarget

## Abstract

Structural Equation Models (SEM) can explicitly distinguish “dementia-relevant” variance in cognitive task performance (i.e., “δ” for dementia). In prior work, δ appears to uniquely account for dementia severity regardless of the cognitive measures used to construct it. In this study, we test δ as a mediator of age's prospective association with future cognitive performance and dementia severity in a large, ethnically diverse longitudinal cohort, the Texas Alzheimer's Research and Care Consortium (TARCC). Age had adverse effects on future cognition, and these were largely mediated through δ, independently of education, ethnicity, gender, depression ratings, serum homo-cysteine levels, hemoglobin A1c, and apolipoprotein e4 status. Age explained 4% of variance in δ, and through it, 11-18% of variance in future cognitive performance. Our findings suggest that normative aging is a dementing condition (i.e., a “senility”). While the majority of variance in dementia severity must be independent of age, age's specific effect is likely to accumulate over the lifespan. Our findings also constrain age's dementing effects on cognition to the age-related fraction of “general intelligence” (Spearman's “*g*”). That has broad biological and pathophysiological implications.

## INTRODUCTION

Cognitive performance is associated with age, and seemingly healthy elderly persons may be impaired relative to young adult norms [1]. Age is also a major risk factor for dementia, whether attributed to Alzheimer's disease (AD) or to other neurodegenerative disorders [2]. However, it is an empirical question whether neurodegenerative changes mediate the association between age and cognition, or indeed, whether age-specific cognitive changes might dementing in their own right.

We do not yet know the phenotype of age-specific cognitive change. Many studies describe longitudinal age-related trends in cognition [3-5]. However, those estimates have significant interindividual variability which suggests the existence of subgroups, any of which might uniquely exhibit the true Aging-Specific Cognitive Phenotype (ASCP).

We have used a Growth Mixture Model (GMM) analysis, to better precise the ASCP [6]. This allowed us to distinguish an “Aging Proper” subgroup, and to characterize its performance on a battery of cognitive measures. Aging Proper was characterized by improving verbal function, stable memory and declines in non-verbal performance and Instrumental Activities of Daily Living (IADL) [7]. In contrast, a presumed pathological “Aging et alia” group was characterized by declines in general cognition, memory, executive function, and IADL. 40.2% of subjects in the Aging Proper group had survived up to a decade after their assessment, compared with 16.9% of subjects the in Aging et alia group. 79.3% of survivors classified as Aging Proper remained in independent levels of care a decade later (at a mean age = 88.8 ± 3.4 years) compared with 53.9% of Aging et alia. However, declines in IADL suggested that both groups may have been experiencing one or more *dementing processes*. If Aging Proper is dementing, then even normative cognitive aging may be a matter of concern, and a potential target for therapeutic intervention.

Meanwhile, we have recently been using a theory driven Confirmatory Factor Analysis (CFA) in a Structural Equation Model (SEM) framework to construct a latent phenotype *for dementia itself*, as distinct from either global cognitive performance or performance in any single cognitive domain [8]. Our intent has been to identify dementia's essential biomarkers, and to distinguish them from those of an illness' non-dementing features. The resulting latent variable, i.e., “δ” (for “dementia”), is relatively free of measurement error, continuously distributed, and appears to be “indifferent” to its cognitive indicators. δ is strongly related to IADL, and with dementia severity, as measured by the Clinical Dementia Rating Scale “Sum of boxes” (CDR) [9-10].

δ is extracted from Spearman's general intelligence factor “*g*” [11]. δ's remainder in *g* is also extracted from our model, and has been labeled “g’ “ to distinguish it from *g* itself. In contrast to δ, g’ is weakly associated with CDR, has a poor Area Under the Receiver Operating Curve (AUC; ROC) for the discrimination between AD cases and controls, and is not associated with IADL (by definition).

We have successfully validated numerous δ homologs in three different cohorts [8, 10, 12-13]. δ has also been independently replicated by a second group in the National Alzheimer's Coordinating Center (NACC)'s Uniform Dataset (UDS) (n = 26,606) [14]. Regardless of either the sample frame or the measures used to construct it, the latent variable δ appears to be strongly related to the CDR and achieves very high AUCs for the discrimination between AD cases and either controls (range 0.987-0.995) or Mild Cognitive Impairment (MCI) (range 0.950-0.957).

It is an empirical question whether age is associated with cognitive performance independently of δ, whether δ and /or g’ mediate(s) their relationship, or whether multiple pathways are involved. These questions can be addressed via mediation models [15]. If δ is found to mediate age's association with prospective cognitive performance, then Aging Proper might join the ranks of potentially dementing conditions.

Longitudinal mediation models are arguably causal [15]. We propose to test δ and g’ as temporally intermediate mediators of baseline age's effect on prospective cognitive performance. This may seem trivial, but it is not. If δ scores characterize the ASCP, then normative aging becomes a dementing condition and its effect may sum independently of other dementia risks to advance the onset of a clinically demented state, and /or modulate the presentations of neurodegenerative disorders *by independent mechanisms*.

If age is an independent determinant of dementia severity, then its effect on δ ought to be independent of comorbidities, including depression (which has previously been shown to affect δ) [12] and neurodegenerative diseases. The biomarkers mediating the association between age and cognition could be determined. They might offer opportunities for the specific remediation, modulation or prevention of age-specific cognitive disability.

## RESULTS

Descriptive statistics are presented in Table 1. The mean age of TARCC participants at baseline was 70.9 (9.7) years, with a range of 52-102 years. There were no cross-group differences between the two randomly selected subgroups (by Student's t, all p >0.05).

**Table 1:** Descriptive statistics

			Student's *t*		
	Variable N	Total Sample	Group 1 *N* = 1544 Mean (SD)	Group 2 *N* = 1528 Mean (SD)	*p*
**Baseline (Wave 1)**					
**Gender (% female)**	3071	61.0	62.0	60.0	0. 50
**Ethnicity (% Hispanic)**	3071	36.0	36.0	37.0	0.62
**Age at Baseline Visit**	3072	70.9 (4.3)	71.1 (9.7)	70.8 (9.6)	0.33
**Education**	3072	13.2 (4.3)	13.2 (4.3)	13.2 (4.3)	0.69
**MMSE**	3071	25.4 (4.9)	25.3 (4.9)	25.6 (4.8)	0.25
**CDR (Sum of Boxes)**	3066	2.5 (3.4)	2.5 (3.5)	2.4 (3.3)	0.12
**GDS (30 item)**	2765	5.6 (5.3)	5.7 (5.2)	5.5 (5.4)	0.53
**Wave 2**					
**LM II**	1882	8.9 (4.9)	8.8 (5.0)	8.9 (4.9)	0.85
**VR I**	2116	8.3 (4.3)	8.3 (4.3)	8.2 (4.2)	0.96
**COWA**	2212	8.7 (3.6)	8.5 (3.7)	8.8 (3.6)	0.08
**DST**	2272	9.0 (3.2)	8.9 (3.2)	9.1 (3.2)	0.39
**IADL (Summed)**	2311	12.2 (6.7)	12.3 (6.8)	12.0 (6.6)	0.17
**Wave 3**					
**Animal**	1504	14.5 (6.4)	14.3 (6.6)	14.6 (6.1)	0.35
**Boston**	1696	8.6 (4.7)	8.4 (4.5)	8.8 (4.5)	0.06
**CERAD**	1159	18.6 (6.5)	18.5 (6.8)	18.7 (6.3)	0.54
**Trails A**	1607	8.4 (3.9)	8.4 (4.0)	8.4 (3.9)	0.98
					

Animal = Animal Naming Test; APOE = apolipoprotein e4 status; Boston = Boston Naming Test; CDR = Clinical Dementia Rating Scale Sum of Boxes; CFI = Comparative Fit Index; CERAD = Consortium to Establish a Registry of Alzheimer's Disease List Learning Total score; COWA = Controlled Oral Word Association Test; DF = degrees of freedom; DIS = Digit Span Test; GDS = Geriatric Depression Scale; GDS = Geriatric Depression Scale; IADL = Instrumental Activities of Daily Living; LM II = Weschler Memory Scale: Delayed Logical Memory; MMSE = Mini-mental State Exam; SD = standard deviation; RMSEA = Root Mean Square Error of Association; SD = standard deviation; S.E. = Standard Error; Trails A = Trail Making Test Part A; VR II = Weschler Memory Scale: Delayed Visual Reproduction.

The unadjusted dEQ composite's AUC for the discrimination between AD cases and controls at Wave 2 was high [AUC = 0.986 (CI: 0.981-0.991)]. g's AUC for the same discrimination was at a near chance level [AUC = 0.630 (CI: 0.601-0.65.9)]. This is consistent with past findings, across batteries, in this and other cohorts.

**Figure 1:**
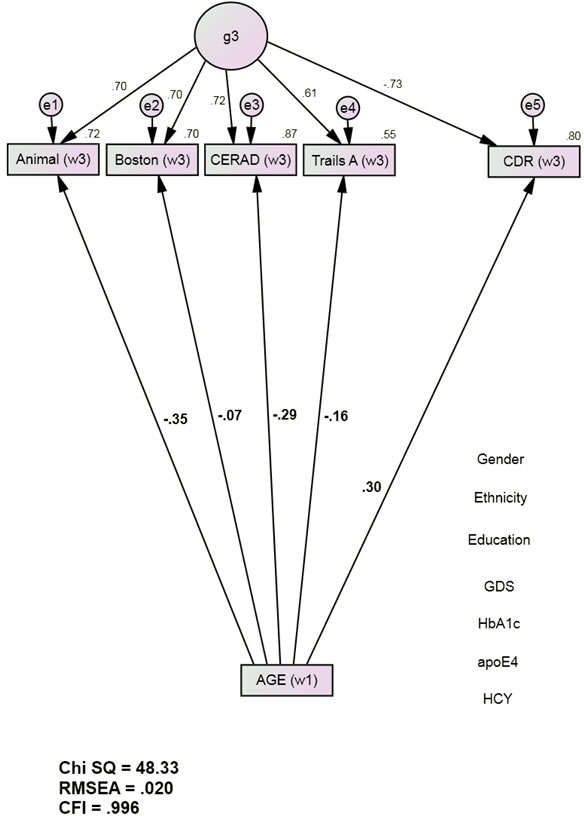
Age's direct associations with future cognition and dementia severity (group 1, *n* = 1544) Animals = Animal Naming Test; APOE = apolipoprotein e4 status; Boston = Boston Naming Test; CDR = Clinical Dementia Rating Scale Sum of Boxes; CFI = Comparative Fit Index; CERAD = Consortium to Establish a Registry of Alzheimer's Disease List Learning Total score; DF = degrees of freedom; GDS = Geriatric Depression Scale; HCY = serum homocysteine; HgbA1c = serum hemoglobin A1c; RMSEA = Root Mean Square Error of Association; SD = standard deviation; S.E. = Standard Error; Trails A = Trail Making Test Part A. *All observed variables except Age are adjusted for education, ethnicity, gender, GDS, HCY, HgbA1c, and APOE e4 status (paths not shown for clarity). The covariates are densely intercorrelated.

The Base Model (Figure 1) had excellent fit [χ^2^ = 48.33 (22), p < 0.001; CFI = 0.996; RMSEA = 0.020]. Baseline Age was significantly associated with Wave 3 cognitive performance and with Wave 3 CDR. Age's effects on Wave 3 cognitive performance were significant and inverse (adverse), ranging from Boston (r = −0.07, p = 0.04) to Animal (r = −0.35, p <0.001) (Figure 1). Age was moderately associated with Wave 3 CDR scores (r = 0.30, p <0.001). The positive association implies an adverse effect on future dementia severity.

**Figure 2:**
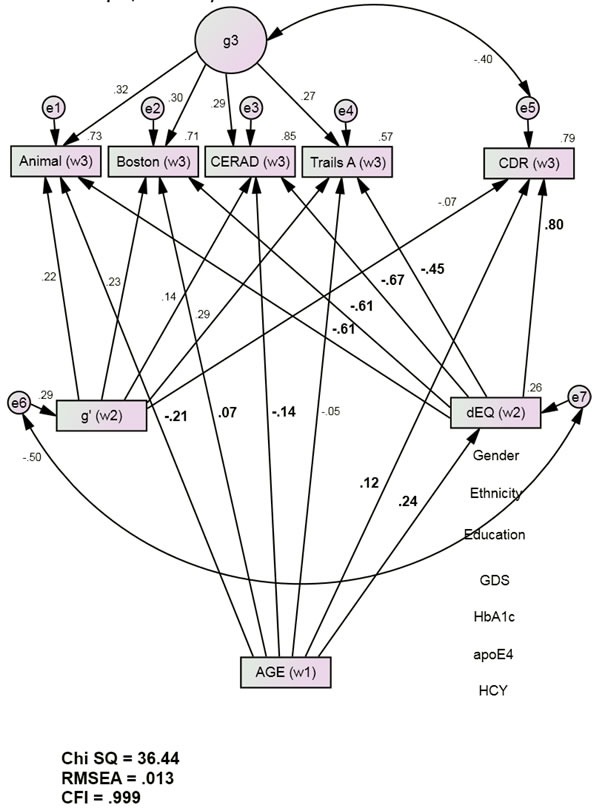
dEQ Mediates Age's Associations with Future Cognition and Dementia Severity (Group 1, *n* = 1544) Animals = Animal Naming Test; APOE = apolipoprotein e4 status; Boston = Boston Naming Test; CDR = Clinical Dementia Rating Scale Sum of Boxes; CFI = Comparative Fit Index; CERAD = Consortium to Establish a Registry of Alzheimer's Disease List Learning Total score; DF = degrees of freedom; GDS = Geriatric Depression Scale; HCY = serum homocysteine; HgbA1c = serum hemoglobin A1c; RMSEA = Root Mean Square Error of Association; SD = standard deviation; S.E. = Standard Error; Trails A = Trail Making Test Part A. *All observed variables except Age are adjusted for education, ethnicity, gender, GDS, HCY, HgbA1c, and APOE e4 status (paths not shown for clarity). The covariates are densely intercorrelated.

The final mediation model (Figure 2) also had excellent fit [χ^2^ = 36.44 (24), p < 0.001; CFI = 0.999; RMSEA = 0.013]. Independently of the covariates (i.e., education, ethnicity, gender, GDS scores, HCY, Hgb A1c, and APOE e4 burden) baseline Age was significantly directly associated with Wave 3 Animal, Boston and CERAD. These direct associations were statistically weak (Boston, CERAD) to moderate (Animals) and attenuated relative to the Base Model. Age's direct association with Wave 3 CDR was also attenuated and now statistically weak (r = 0.12, p <0.001). Age was significantly associated with Wave 2 dEQ (r = 0.24, p < 0.001), but not with the Wave 2 g’ composite (r = 0.00, p = 0.87). Age's significant association with Wave 2 dEQ scores was moderately strong and in a positive direction (r = 0.24, p <0.001). Given Age's unstandardized association of 0.02 with dEQ, each year of increasing age is associated with an 0.02 SD increase in dEQ.

Because age was not significantly associated with g’, it cannot be a mediator of Wave 3 cognitive performance. g’ was therefore omitted from further consideration. Independently of both Wave 2 g’ ‘s adverse effects, and Wave 3 g's, Wave 2 dEQ was strongly inversely related to Wave 3 cognitive performance (and positively with CDR). Thus, the age-related effects mediated by dEQ were functionally adverse and likely to reinforce Age's attenuated and relatively weak direct effects.

Age's significant direct effects in Model 1 were largely or fully mediated by dEQ (Table 2). Similar results are obtained if alternative measures are substituted. For example, 91.4% of Age's effect on Wave 3 MMSE scores were mediated by dEQ, while Age's effects on Wave 3 LMII performance were fully mediated (data not shown).

**Table 2:** dEQ's mediation effects (group 1)

Path	Description	Unadjusted (Figure 1)	Adjusted (Figure 2)	%Mediation
**a**	Age > Animalw3	−0.35, *p* < 0.001	−0.21, *p* = 0.955	42
**b**	Age > Bostonw3	−0.07, *p* = 0.037	0.07, *p* = 0.013	91
**c**	Age > CERADw3	−0.29, *p* < 0.001	−0.14, *p* = 0.006	54
**d**	Age > TrailsAw3	−0.16, *p* < 0.001	−0.05, *p* < 0.001	70
**e**	AGE > CDRw3	0.30, *p* < 0.001	0.12, *p* < 0.001	86

Animal = Animal Naming Test; Boston = Boston Naming Test; CDR = Clinical Dementia Rating Scale Sum of Boxes; CERAD = Consortium to Establish a Registry of Alzheimer's Disease List Learning Total score; Trails A = Trail Making Test Part A.

The constrained model's fit was statistically indistinguishable from the unconstrained model. Therefore age's associations with all Wave 2 and Wave 3 measures remain stable across both subsamples (Table 3).

In post hoc analyses, Age's direct association with dEQ was statistically indiscriminable in the young old (r = 0.17, p <0.001) vs. the elder old (r = 0.11, p <0.001) [χ^2^ difference = 0.09 (1), p = 0.75]. Nor was it affected by a diagnosis of dementia (AD: r = 0.09, p <0.001; MCI + NC: r = 0.09, p <0.001) [χ^2^ difference = 0.01 (1), p = 0.90].

**Table 3:** cross-group validation of the mediation model (Figure 2)

Model	df	Χ2	Χ2 difference	*p*
Base Model	24	36.44		
Constrained	30	41.54	5.10 (6)	0.50

df = degrees of freedom

## DISCUSSION

We have used a formal, longitudinal mediation analysis to test a δ homolog as a mediator of age's effects on observed cognitive performance. The analysis has many strengths. Our sample size is large and ethnically diverse. The model is longitudinal, has excellent fit, and was replicated across two large randomly selected subsamples. There is no overlap in the cognitive measures used to construct the Wave 2 mediators and the outcomes used to test the mediation effects. The dEQ mediator achieved a high AUC for AD's diagnosis. The entire model is adjusted for multiple competing sources of cognitive variance.

We have two principal findings. First, age's adverse effects on cognition were largely mediated through δ. Because δ specifically mediates cognition's association with disability, Age's contributions along this path are potentially “dementing”, and can be expected to modulate age-independent causes of dementia. Second, Age accounts for little variance in dEQ (5%) and by that path accounts for a relatively small fraction future outcomes, ranging from R^2^ = 0.11 (Age > dEQ > Trials A) to 0.16 (Age > dEQ > CERAD). Age-independent mechanisms must therefore be the dominant determinants of the dementia risk of older persons.

We found that age's adverse effects on cognition were largely mediated through δ. Age had incremental effects independently of δ, but these were statistically weak. dEQ's residual in Spearman's general intelligence factor “*g*” (i.e., g’) represents an additional influence on observed cognitive performance. It risks to obscure Age's specific effects. However, 1) g’'s contribution, even in older persons, *is not age-related*, and 2) g’'s contribution is not functionally salient (by definition).

In contrast to g’, future dementia severity has been shown to be almost entirely attributable to δ and its rate of change in time (Δδ) [14, 16]. Therefore, any predictor of δ scores might have clinical significance, even age's weak effect.

Since age's δ-mediated effects explain a substantial fraction of its total effect on cognitive performance, age largely exerts a disabling and therefore potentially dementing cognitive influence. Since its contribution to δ is also stable across diagnostic subgroups, and independent of multiple cormorbidities, age must be dementing in its own right (i.e., an age-specific dementia = “senility”).

Age's contribution to δ is also stable regardless of whether it is measured across TARCC's entire sample, or across younger and older subgroups. This suggests that age's effect is likely to accumulate linearly over the lifespan. Given that each year of increasing age is associated with an 0.02 SD increase in dEQ, 1.0 SD in dEQ scores might be traversed over TARCC's 50 year age range due to aging alone. This is not trivial. We have observed that each *quintile* increase in the dEQ scores of cognitively normal persons increases their five year risk of dementia conversion by 50%, while the risk of cases with “Mild Cognitive Impairment (MCI)” increases three-fold [17].

Age's independent contribution should be maximal then among the oldest old (e.g., centenarians). This could explain 1) the weakened association between APOE and dementia in centenarians [18-20], despite dementia's increasing incidence [21], and 2) the reduced burden of neurodegenerative lesions among the oldest “AD” cases [22].

Age's cumulative effects on δ scores over a lifetime will be more likely to result in clinical “dementia” if they are added to a less advantaged baseline. Thus, youthful cognitive performance can be associated with late onset dementia, while youthful educational attainment seems to protect one from it [23-24]. This might also explain the propensity of remote insults, such as traumatic brain injury (TBI) to increase dementia risk, despite intervening decades of normal performance.

Age is significantly associated with δ in the NACC, and their association is entirely mediated by AD neuropathology in autopsy proven AD cases [25]. However, age's association with AD pathology was inverse, suggesting that older age was associated with dementias exhibiting a lesser burden of AD pathology. This suggests that the adverse effect of age on cognition may not be mediated by the Alzheimer process, but by its direct effects on δ.

A distinct tauopathy, limited to the hippocampus and entorhinal cortex and with no more than minimal Aβ deposition, has been proposed to represent the “primary age-related tauopathy (PART)” [26]. PART is almost universally present in extremely old individuals. It would be interesting if PART pathology mediated age's association with δ. However, δ has been related instead to the Default Mode Network (DMN) [12, 27]. The DMN's involvement by tauopathy may be necessary to dementia's manifestation in AD [28]. PART pathology is not so situated, and may therefore not explain dementia in older persons [29].

We have recently constructed a δ “ortholog” representing age-specific cognitive performance (i.e., “cAGE”) [30]. That ortholog had a similar bifactor structure to dEQ's, overlapping cognitive indicators and identical covariates, but targeted age instead of IADL. Similarly to this model, cAGE adversely affected cognitive performance independently of HCY, HgbA1c, and APOE.

Serum insulin-like growth factor binding protein 2 (IGF-BP2) was strongly associated with cAGE and, through it, appeared to adversely affect cognition. IGF-BP2 attenuates Age's effect on dEQ in Model 2 (DRR Unpublished), and may therefore be a partial mediator of age's specific effect on δ. IGF-BP2 is not a serum biomarker of *age adjusted* δ scores. TARCCs remaining biomarker panel is being explored for other biomarkers of age-specific cognitive change.

In other recent work we have suggested that activation of the Toll-Like Receptor (TLR) may contribute to δ scores [31]. Although TLR function appears to be dysregulated in old age [32], our finding was age adjusted, and may therefore represent an age-independent determinant of δ scores. δ's role as a potential mediator of age-independent dementia risks is beyond the scope of this analysis.

Our model has yet to be replicated in an independent sample. However, it survives generalization across two large random subsets of TARCC's cohort. Replication might be feasible in the NACC. The NACC dataset has the advantage of including several dementia etiologies and associated neuropatholgy. If age contributes to δ scores independently of multiple pathologies, then age's apparent role as a risk factor for those dementias could be called into question. Instead, age's effects on δ could then be interpreted as an *independent comorbid dementing process*.

Our findings underscore the potential significance of a dementing ASCP in a rapidly aging demographic. Late Onset “AD” (LOAD) cases are by far the most common manifestation of dementia, and octogenarians are the fastest growing demographic segment of the western world, including the U.S. [33]. SEM analyses of large datasets provide an approach to the disaggregation of age-specific influences over cognitive performance. Only then can the biomarkers of Aging Proper be identified, and steps taken to understand its specific influence on dementia risk.

## MATERIALS AND METHODS

### Subjects

#### Texas Alzheimer's Research and Care Consortium (TARCC)

The Consortium's methods have been described in detail elsewhere [34]. Briefly, the TARCC cohort is a convenience sample of n = 3069 well characterized cases of Alzheimer's disease (AD) (n = 1182), “MCI” (n = 611), and normal controls (NC) (n = 1276). Each TARCC participant undergoes a standardized annual examination that includes a medical evaluation, neuropsychological testing, and clinical interview. Diagnosis of AD status is based on National Institute for Neurological Communicative Disorders and Stroke-Alzheimer's Disease and Related Disorders Association (NINCDS-ADRDA) criteria [35]. Institutional Review Board approval was obtained at each site and written informed consent was obtained for all participants. DRR is PI of the San Antonio TARCC site, and a member of TARCC's Steering Committee.

This analysis was performed on data obtained prior to 2015. We divided the cohort into two randomly selected subsets. Group 1 (n = 1544) was used to construct the model. Group 2 (n = 1528) was used to replicate the parameters of interest (i.e., the associations between baseline age, Wave 2 δ and g’, and Wave 3 cognitive performance).

#### δ's Wave 2 Indicators

Logical Memory II [36]: Following a thirty minute delay, the subject recalls two paragraphs read aloud. Delayed paragraph recall has been useful clinically in identifying dementia and tracking progression of the disease.

Visual Reproduction I [36]: The subject recalls two paragraphs read aloud. Visual Reproduction has been useful clinically in identifying dementia and tracking progression of the disease.

The Controlled Oral Word Association (COWA) [37]: The COWA is a test of oral word production (verbal fluency). The patient is asked to say as many words as they can in one minute, beginning with a certain letter of the alphabet.

Digit Span Test (DST) [36]: The DST sums the longest set of numbers the subject can repeat back to the examiner in correct order (forwards and backwards).

Instrumental Activities of Daily Living (IADL) [7]: IADL's were assessed using informant ratings. The ability to use the telephone, shopping, food preparation, housekeeping, laundry, use of transportation, ability to handle finances, and responsibility for medication adherence were each rated on the Likert scale ranging from 0 (no impairment) to 3 (specific incapacity). A total IADL score calculated as the sum of all eight items.

### Wave 3 Outcome Variables

Animal Naming (Animals) [38]: This test of verbal fluency asks the subject to produce as many animal names as they can in one minute.

The Boston Naming Test (BOSTON) [39]: This is a confrontation naming test that requires the subject to verbally name each of 60 line drawings of objects of increasingly low frequency.

Consortium Establish a Registry for Alzheimer's Disease List Learning (CERAD) [40]: This memory test uses a list of ten unrelated words that the subject reads out loud over three learning trials to ensure registration. Following a three to five minute delay, a free recall trial and a recognition trial are presented.

Trail-Making Part A (Trails A) [41]: This test of attention, speed and mental flexibility requires the subject to connect numbers, placed randomly on the page, in their appropriate order.

### Clinical Covariates

Education: Education was coded continuously as years of formal education.

Ethnicity: Ethnicity was determined by self-report and coded dichotomously as “Hispanic” and “non-Hispanic”. All TARCC evaluations and psychometrics are provided in English or in Spanish according to the subject's preference.

### Gender: Gender was coded dichotomously

The Geriatric Depression Rating Scale (GDS) [42-43]: GDS scores range from zero-30. Higher scores are worse. A cut-point of 9-10 best discriminates clinically depressed from non-depressed elderly.

### Biomarker Covariates

#### Laboratory Analysis

Non-fasting samples were collected, centrifuged and pipetted by 1mL aliquots into polypropylene cryovial tubes and placed in −200°C (non-frost free) or −80°C freezers until shipment to the TARCC Biobank.

Measurements of homocysteine (HCY), hemoglobin A1c (HgbA1c), and apolipoprotein (APOE) ε4 genotyping were performed in the Ballantyne laboratory at the Baylor College of Medicine. HgbA1c was measured in whole blood. HCY was measured in serum. HgbA1c measurement was based on the turbidimetric inhibition immunoassay (TINIA) for hemolyzed whole blood. HCY concentrations were measured using the recombinant enzymatic cycling assay (i.e., Roche Hitachi 911).

APOE genotyping was conducted using standard polymerase chain reaction (PCR) methods [44]. APOEε4 status was coded 0-2 according to the number of ε4 alleles.

### Clinical Correlates

The Clinical Dementia Rating Scale sum of boxes (CDR) [9]: The CDR is used to evaluate dementia severity. The rating assesses the patient's cognitive ability to function in six domains –memory, orientation, judgment and problem solving, community affairs, home and hobbies and personal care. The information necessary to those ratings is collected during an interview with the patient and their caregiver. Each domain is rated on a scale of 0.0-3.0. A total CDR-SB is calculated as the sum of all six domains.

The Mini-Mental Status Examination (MMSE) [45]: The MMSE is a well-known and widely used test for cognitive impairment screening. Scores range from 0 to 30. Scores less than 24 reflect cognitive impairment.

### Statistical Analyses

#### Analysis Sequence

Confirmatory factor (CFA) and mediation models were performed using Analysis of Moment Structures software (AMOS) [46]. The maximum likelihood estimator was chosen for these models. δ and g’ were derived by bifactor CFA from baseline data.

A δ homolog was constructed from Wave 2 data. This is structurally identical to “dEQ”, which has elsewhere been reported to 1) have excellent fit (i.e., χ^2^/df = 181/24, p < 0.001; CFI = 0.97; RMSEA = 0.05), 2) have acceptable factor determinacy by Grice's Method [47], 3) exhibit factor equivalence across ethnicity, 4) to be strongly correlated with dementia severity as measured by the CDR (r = 0.99, p <0.001) and 5) to exhibit an AUC of 0.95 (CI = 0.94 - 0.96) for the discrimination between AD cases and controls (in Wave 1 TARCC data) [31]. However, for the purposes of this analysis, dEQ was constructed without any covariates, specifically age, HCY, HGbA1c and APOE e4 burden.

dEQ and g’ factor weights were applied to Wave 2 observed data to generate Wave 2 dEQ and g’ composite scores (i.e., dEQ w2 and g’ w2, respectively). g’ is dEQ's residual in Spearman's *g*. The composite scores were used as observed mediators of baseline age's direct associations with Wave 3 CDR scores (CDR w3) and Wave 3 cognitive performance. There is no overlap in δ's Wave 2 indicators and selected Wave 3 outcome measures.

In the resulting nested longitudinal models, dEQ and g’ are being tested as competing independent mediators of age's direct effects on prospective cognitive performance. All observed Wave 2 and Wave 3 measures were adjusted for education, ethnicity, gender, GDS scores, HCY, Hgb A1c, and APOE ε4 status.

Both the Wave 1 dEQ homolog and the mediation model were constructed in a randomly selected subset of TARCC participants, comprising approximately 50% of the subjects (i.e., Group 1: n = 1544). As a test of the model's generalizability to the remainder (n = 1528), age's significant direct associations were constrained across the two groups, and model fit compared across constrained and unconstrained conditions.

In post hoc analyses, we further ascertained the effects of stratification at 79/80 years on Age's direct association with dEQ (“young elderly” mean age = 67.47, n = 2428; “older elderly” mean age = 83.93, n = 644). We also tested the effects of a dementia diagnosis [“AD”: n = 1182 vs. “MCI” OR NC: n = 1887].

These models were all constructed in an SEM framework, using raw data. No statistical transformation was applied to biomarker data. Modern Missing Data Methods were automatically applied by the AMOS software (see below).

### Mediation models

Following the approach of MacKinnon et al. [48], we first modeled “direct” paths between baseline Age and Wave 3 clinical outcomes. We next tested each Wave 2 composite as an independent predictor of the Wave 3 clinical outcomes, in separate models. If the composite in question was also significant, we then tested the indirect path as a mediator of the “direct” path(s). Figure 2, for example, presents the mediation model for dEQ.

According to Kraemer et al. [15], the timing of the variables relative to each other is important. For a true mediation effect, the mediator should occur between the predictor and the outcome, not coincidentally with the predictor. Our model is constructed across three annual assessments, with Wave 2 composites serving as temporally intermediate mediators of baseline age's association with Wave 3 clinical outcomes. The results can therefore be interpreted causally.

### Missing data

888 subjects had complete biomarker data. In contrast, psychometrics and adjudicated clinical diagnoses were available on 2017 subjects. Rather than using case-wise deletion, the missing biomarker and psychometric data were handled by Modern Missing Data Methods [49].

Only the ROC analyses, which were performed in Statistical Package for the Social Sciences (SPSS) [50] were limited to complete cases. AMOS employs Full information Maximum Likelihood (FIML) methods to address missing data. FIML uses the entire observed data matrix to estimate parameters with missing data. FIML yields unbiased parameter estimates, preserves the overall power of the analysis, and is arguably superior to alternative methods, e.g., multiple imputation [51-52].

### Fit indices

Model fit was assessed using four common test statistics: chi-square, the comparative fit index (CFI), and the root mean square error of approximation (RMSEA). Where two nested models were compared, Akaike's Information Criterion (AIC) was added. A lower AIC statistic indicates better fit. A non-significant chi-square signifies that the data are consistent with the model [53]. However, in large samples such as TARCC, this metric is limited by its tendency to achieve statistical significance when all other fit indices (which are not sensitive to sample size) show that the model fits the data very well. A CMIN/DF ratio < 5.0 suggests an adequate fit to the data [54]. The CFI statistic compares the specified model with a null model [55]. CFI values range from 0 to 1.0. Values below 0.95 suggest model misspecification. Values approaching 1.0 indicate adequate to excellent fit. An RMSEA of 0.05 or less indicates a close fit to the data, with models below 0.05 considered “good” fit, and up to 0.08 as “acceptable“[56]. All fit statistics should be simultaneously considered when assessing the adequacy of the models to the data.

### ROC curves

The diagnostic performance or accuracy of a test to discriminate diseased from normal cases can be evaluated using ROC curve analysis [57-58]. Briefly the true positive rate (Sensitivity) is plotted as a function of the false positive rate (100-Specificity) for different cut-off points of a parameter. The area under the ROC curve (AUC) is a measure of how well a parameter can distinguish between two diagnostic groups (diseased/normal). The ROC analysis was performed in SPSS.
